# Synthesis of Bimetallic Platinum Nanoparticles for Biosensors

**DOI:** 10.3390/s130810358

**Published:** 2013-08-12

**Authors:** Gerard M. Leteba, Candace I. Lang

**Affiliations:** 1 Centre for Materials Engineering, Department of Mechanical Engineering, University of Cape Town, Private Bag, Riondebosch 7701, South Africa; E-Mail: gerard.leteba@uct.ac.za; 2 Department of Engineering, Macquarie University, Sydney, NSW 2109, Australia

**Keywords:** biosensors, bimetallic nanoparticles, magnetic nanoparticles, platinum nanoalloys, Pt-Ni, Pt-Co, Pt-Fe

## Abstract

The use of magnetic nanomaterials in biosensing applications is growing as a consequence of their remarkable properties; but controlling the composition and shape of metallic nanoalloys is problematic when more than one precursor is required for wet chemistry synthesis. We have developed a successful simultaneous reduction method for preparation of near-spherical platinum-based nanoalloys containing magnetic solutes. We avoided particular difficulties in preparing platinum nanoalloys containing Ni, Co and Fe by the identification of appropriate synthesis temperatures and chemistry. We used transmission electron microscopy (TEM) to show that our particles have a narrow size distribution, uniform size and morphology, and good crystallinity in the as-synthesized condition. Energy dispersive spectroscopy (EDS) and X-ray diffraction (XRD) confirms the coexistence of Pt with the magnetic solute in a face-centered cubic (FCC) solid solution.

## 1. Introduction

Considerable attention has been paid to the potential of nanoalloys for biosensors, even though synthesis of these nanoparticles presents unresolved problems. Metallic nanoparticles show promise in many biosensing applications including as carriers for biological components [1], as magnetic probes for magnetoresistance biosensors [2,3] and in bioassays [2,4]. *Magnetic* nanoparticles allow for magnetic separation and magnetic collection of biomaterials, such as recovery of gene products within cells [5]; but the use of *metallic* nanoparticles for these applications is not yet widespread [6]. This is, in part, due to the sensitivity of the magnetic metals—iron, cobalt and nickel (Fe, Co and Ni)—to oxidation, which diminishes their magnetic properties [7,8]. A route to avoiding this difficulty lies in the addition of a second metallic element to produce bimetallic nanoparticles (nanoalloys); ideally this increases the oxidation resistance while maintaining magnetic properties. Multimetallic nanoparticles have already been the subject of considerable interest in the development of novel nanoparticles [9–11], but to date they have not been extensively investigated for biosensors. Platinum-based bimetallic nanoparticles, with solute magnetic elements Ni, Co and Fe, can exhibit enhanced magnetic anisotropy and chemical stability in contrast to monometallic Ni, Co and Fe nanoparticles [12,13]; furthermore, the catalytic activity of platinum alloys (such as Pt-Co, Pt-Ni and Pt-Fe) could enhance the detection limits of biosensors [14]. Bimetallic platinum nanoparticles are accordingly emerging as exciting candidates for the development of biosensors.

The need for more reliable, tailored synthetic protocols for magnetic nanoparticles has been noted by Jun *et al.* [15], as a prerequisite for development of the next generation of magnetic nanodevices in biological technologies. This need arises from the widely-reported observation that the properties of nanoparticles are sensitively dependent on their size and shape, which in turn are controlled by the synthetic process which is used. For instance, the properties of cobalt nanoparticles are particularly sensitive to size: Park *et al.* [16] report that their magnetic coercivity reduces significantly above 10 nm. This type of sensitivity offers the potential to manipulate properties by manipulating nanoparticle size, using the appropriate synthesis protocol; but it also signifies that if synthesis is not tightly controlled, then properties may be at considerable variance with what is anticipated. To complicate matters further, the precise nature of the synthesis-structure-property relationship is often poorly understood for multimetallic nanoparticles, so that reproducible size, shape and properties are not always achieved [17].

The preparation of magnetic nanoparticles for use in biological sensors requires a balance between desirable sensor properties and low biological toxicity. The Pt-M nanoalloys (M = 3d transition element, magnetic) are outstanding candidates for these applications; however fabrication of nanoscale magnetic alloys with a narrow size distribution, uniform shape and controlled composition remains a key challenge. Although magnetic nanoalloys can offer high saturation magnetization [18] and resistance to oxidation [19], careful control of composition, size and shape must be maintained in order to achieve optimal properties. Examples of sensitivity to composition include the Co-Pt alloy which is an outstanding permanent magnet at the 1:1 stoichiometry if suitably heat-treated [20]; and FePt_3_, a room-temperature ferromagnet which, at the 1:3 stoichiometry, compares favorably with other ferromagnetic nanomaterials [21].

**Scheme 1.**
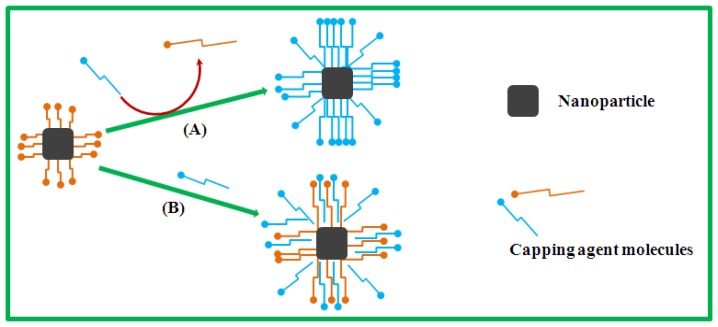
Illustration of nanoparticle surface functionalization: (**A**) surfactant-exchange and (**B**) surfactant addition.

An important step for biocompatibility of candidate nanoalloys is to ensure that they are water-soluble; effective functionalization protocols to achieve this may be required. High-quality magnetic nanoalloys are optimally synthesized in nonpolar solvents, resulting in the formation of hydrophobic nanoparticles. The hydrophobicity of such nanoparticles requires further surface modification to enhance their water-solubility and biocompatibility for deployment in biosensing applications [22–28]. The bimetallic nanoparticles reported in the present work were first stabilized by nonpolar organic surfactants, and so for biosensing require the subsequent modification of their hydrophobic surfaces (which only allow dispersion in toluene, hexane and other nonpolar solvents). For biocompatibility requirements, such nanoalloys must be water-dispersible in a pH range of about 5–9, at sodium concentrations of up to a few hundred mM and operating temperatures up to 95 °C [22,23]. The transformation of hydrophobic nanoparticles into hydrophilic nanoparticles can be achieved via two approaches: surface surfactant-exchange and surfactant addition [22–28], as depicted in Scheme 1.

The surface surfactant-exchange (Scheme 1, path (A)) engages the excess addition of water-soluble surfactant in colloidal solution, resulting in the displacement of the original hydrophobic surfactants on the surface of the nanoparticles and thus rendering them water-dispersible. The surfactant addition (Scheme 1, path (B)) involves the interaction of hydrophobic and hydrophilic surfactants on the nanoparticle surface, forming a double-layer structure which is water-soluble functionalized by the incoming hydrophilic surfactants. However, the degree of solubility of such functionalized nanoparticles is determined by the chemical property of the added surfactant. The surface surfactant-transformation process to form hydrophilic from hydrophobic nanoparticles using these two functionalization approaches has shown that the nanoparticles have great potential for use in biosensing applications [22–28].

The chemical synthesis of bimetallic nanoparticles of Pt with highly reactive non-noble solute metals offers particular synthetic difficulties [29]. The present work investigates and identifies optimal synthesis of highly monodisperse hydrophobic Pt-Ni, Pt-Co and Pt-Fe alloy nanoparticles with fine-tuned size and uniform size distribution, shape and composition, using a single (and simple) synthetic preparation protocol. When Pt-based bimetallic nanoparticles are synthesized by the chemical co-reduction of two kinds of metal inorganic precursor salts, the degree of chemical reduction is significantly influenced by the reaction temperature and the reducing agents used. In the present work, surface active agents are employed to manipulate particle growth and direct shape evolution, stabilize the particles and limit the degree of nanoparticle oxidation. The fabrication route reported here can be an effective tool for large-scale production of metallic nanoalloys.

## 2. Experimental Methods

Platinum-based bimetallic nanoparticles in the size range 5–9 nm were successfully synthesized by the simultaneous reduction of Pt and M precursor salts in a homogeneously mixed solution of surfactants. The degree of reduction was manipulated both by temperature and by the reducing agent employed; the reaction time was 60 min in each case.

### Chemicals

The synthetic method employed the following metal precursors from Sigma-Aldrich (Johannesburg, South Africa): chloroplatinic acid (H_2_PtCl_6_, 8% in water), nickel(II) acetate tetrahydrate (≥98%), cobalt(II) acetate tetrahydrate (99.99%) and iron(III) acetylacetonate (≥99.9%). The surfactants were oleylamine (OAm, 70%), trioctylamine (TOA, 98%), octadecylamine (ODA, 90%), and oleyl alcohol (OA, 85%). The reducing agent and solvent were tetrabutylammonuim borohydride (TBAB, 98%) and benzyl ether (BE, 99%), respectively. Solvents such as anhydrous ethanol, acetone and toluene, used for precipitating and cleaning the particles, were all of analytical grade. All the chemicals were used as-received without any further purification.

### Synthesis of Pt-M nanoparticles

A detailed synthetic protocol is described here. A sample of chloroplatinic acid (0.04 g, H_2_PtCl_6_, received as 8% in water and dried) and a 3d metal precursor salt (0.024 g, nickel(II) acetate tetrahydrate; cobalt(II) acetate tetrahydrate or iron(III) acetylacetonate), together with surfactants OAm (15 mL) or OA (15 mL), TOA (15 mL) and ODA (2.4 g) were dissolved in BE (20 mL), a high boiling point solvent, by sonication for 20–30 min. The resulting mixture was then heated to 150 °C and held at that temperature for 5 min under vigorous stirring. The resultant solution was added to the reducing agent TBAB (0.05 g) in a round-bottomed flask; the solution was then heated to 220 °C (Pt-Ni and Pt-Co) or 260 °C (Pt-Fe) and held at that temperature for 60 min.

The final step described above resulted in reduction of the metal precursor salts; the colloidal solution thus produced, was allowed to cool to room temperature followed by the addition of excess ethanol and acetone to flocculate the particles. The isolation-purification process was performed three times to ensure elimination of any unwanted solvent and excess surfactants. The black product was dried and finally re-suspended in 4 mL of toluene by mild sonication, forming a brown colloidal suspension. The particles that were obtained in this way required no size-selection processing.

### Characterization of Pt-M nanoparticles

Samples for transmission electron microscopy (TEM) characterization were prepared by placing a drop of colloidal nanoparticles, re-suspended in a solvent, on a carbon-supported copper grid and allowing the solvent to evaporate either under ambient temperature for at least 2 h, or by using a drying lamp for 30 min. A TECNAI F20 transmission electron microscope (TEM), Electron Microscope Unit, University of the Western Cape, Cape Town, South Africa, operating at 200 keV was deployed for high-resolution (H-R) TEM imaging, selected-area electron diffraction (SAED) and energy-dispersive X-ray spectroscopy (EDS) data collection. Bright-field TEM images were also acquired using a TECNAI T20 TEM. The size distribution of the particles was determined from multiple randomly selected areas of bright-field TEM images, by measuring many individual particle diameters and using Image J analysis [30]. X-ray diffraction patterns were collected with a Philips Huber MC 9300 X-ray powder diffractometer (XRD), Department of Chemistry, University of Cape Town, Cape Town, South Africa, with a CuKα1 radiation source (λ = 1.5406 Å). The operating voltage and current were kept at 48 keV and 30 mA, respectively. Samples for XRD investigation were prepared by mixing a black dried product of nanoparticles with paroton oil, and depositing this randomly on an XRF microfine mylar polyester film on a flat sample holder.

## 3. Results and Discussion

It is generally accepted that the evolution of nanoparticles in a wet chemical synthesis system involves two stages: a nucleation process, and the subsequent Ostwald ripening growth of the nuclei [31,32]. In the nucleation stage, the core driver for the development of atom clusters into seeds is the supersaturation of the metal precursors; whereas in the growth stage, the surface energy of individual crystallographic faces [33] dominates growth, and hence the energy difference between faces gives rise to the evolution of nanoparticle shape. When the nucleation burst is rapid and short, most metal precursors are consumed during nucleation, resulting in deficient feedstock for the growth phase [32]; the final nanoparticle shape is in this case determined only by the nucleation stage, resulting in small and irregular colloids. Both the nucleation stage and the growth stage thus offer the possibility of thermodynamic and kinetic manipulation, with the aim of optimizing the final size and shape of nanoparticles.

The production of multimetallic nanoparticles adds further variables to the synthesis: the nucleation and growth processes of such nanoparticles are difficult to manipulate due to the typically distinct thermodynamic and kinetic characteristics of different metals; the ultimate composition of the alloy nanoparticle is accordingly challenging to regulate [34]. The synthesis of Pt-based alloy nanoparticles with highly reactive solutes such as Ni, Co and Fe thus offers particular difficulties in preparation: because of a large difference in the driving mechanism for reduction of the Pt and M precursors, simultaneous reduction may be elusive. In order to circumvent this, the nucleation and growth stages must be well controlled to provide feedstock for stable Ostwald ripening and also to avoid the spontaneous formation of separate Pt and M monometallic nanoparticles.

**Scheme 2.**
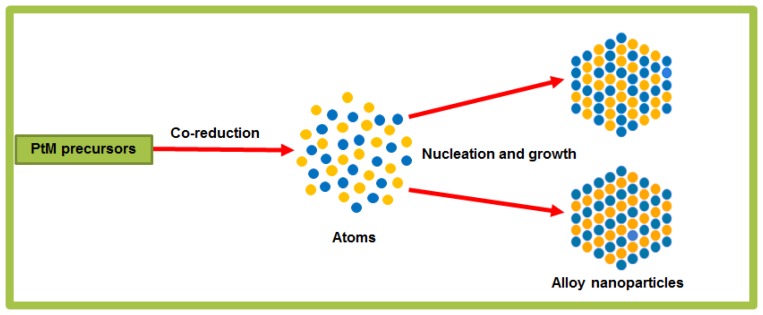
Illustration of synthesis of multimetallic alloy nanoparticles from metal precursors by the co-reduction method.

In the present work, we introduced a strong reducing agent (TBAB) and high temperatures together with a high-boiling-point solvent (BE), to accelerate the reduction of the solute-metal salts and thus to ensure that the kinetics of the reduction of Pt and M precursors are similar. The solvent BE was chosen in this synthetic strategy in order to provide a medium for complete nucleation, growth and interatomic diffusion of both types of metallic atom. A mixture of stabilizing agents, such as OAm, TOA, ODA and OA was used during synthesis in order to regulate the size, dispersion and provide anisotropic growth of alloy nanoparticles. As illustrated in Scheme 2, the synthesis of alloyed nanoparticles in solution-phase by the co-reduction of different metal precursors ideally results in an ordered or disordered arrangement of the different atoms in each nanoparticle, rather than separate nanoparticles of each metal. Our wet chemistry synthetic strategy takes into consideration all the critical reaction parameters for the uniform size and bimetallic compositional control of nanoparticles. Thus, in our preparation route of choice for the synthesis of alloy nanoparticles; the nucleation, growth process and composition of bimetallic nanoparticles were controlled by regulating reaction parameters such as the molar ratio between metal precursors and surface active agents (surfactants), the reaction temperature and time, and reducing agents.

Different bimetallic nanoparticles of Pt-Ni, Pt-Co and Pt-Fe were accordingly synthesized by the single simultaneous reduction of Pt and M metal precursor salts in the presence of surfactants such as OAm, TOA, ODA or OA with TBAB; at 220 °C for Pt-Ni and Pt-Co, and at 260 °C for Pt-Fe; in high-boiling point solvent BE. The higher temperature used for Pt-Fe shortened the reaction time from two hours (at 220 °C) to one hour, consistent with the reaction time of Pt-Ni and Pt-Co. The reduction of both metal ions appeared to be complete after 60 min of heating for all mixtures, forming a dark-brown colloidal solution. This high-temperature reduction route yielded highly monodisperse bimetallic nanoparticles less than 10 nm in size.

**Figure 1.**
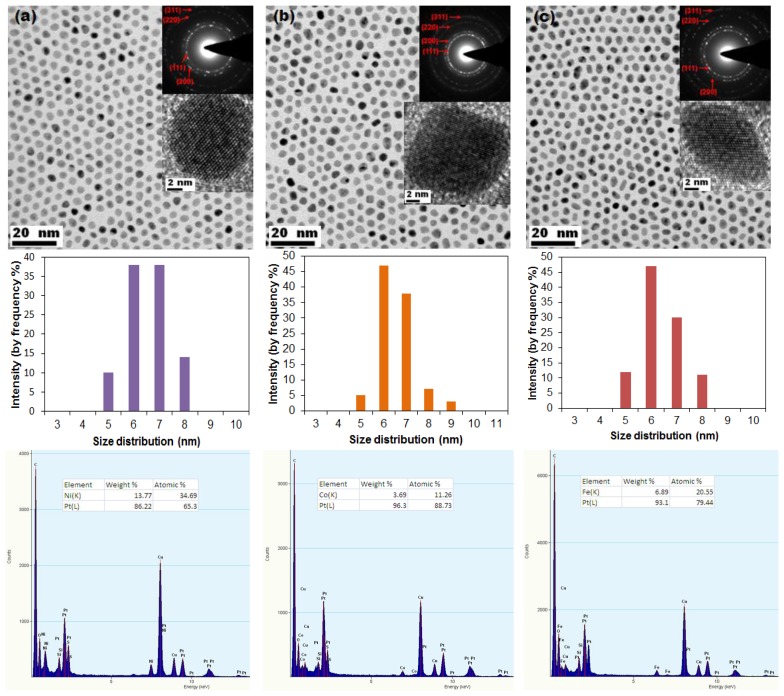
Bright-field TEM images of (**a**) PtNi (**b**) PtCo and (**c**) PtFe nanoparticles synthesized by the simultaneous reduction of metal precursor salts using TBAB as the reducing agent in the presence of OAm, TOA and ODA in BE. The inserts show the FCC electron diffraction patterns and HRTEM images from the corresponding samples. Also shown are the corresponding particle size distribution histogram and EDS spectrum of the nanoalloys.

Figure 1 shows bright-field TEM images of Pt-M nanoparticles, with corresponding particle size distribution histograms and EDS spectra showing composition, for particles synthesized using OAm, TOA and ODA as surfactants. TEM images show monodisperse nanoparticles, with mean diameters in the size range of 6–7 nm, arranged in a two-dimensional hexagonal close-packed array which demonstrates the uniformity of the particle size (in all these EDS spectra: copper (Cu) arises from the metallic support grid whereas carbon (C), silicon (Si) and sulphur (S) arise from the particular carbon support films used in this study). The presence of detectable oxygen suggests that minor oxidation of these binary nanoalloys may have occurred during synthesis, cleaning or preparation of TEM specimens. The described reduction method produced spherical nanoparticles, except for Pt-Fe where there is a combination of both lozenge-shaped and spherical morphologies. In Figure 1, the inserts show SAED patterns and HR-TEM images of the nanoparticles. The rings observed in SAED patterns correspond to the (111), (200), (220) and (311) face-centred cubic (FCC) planes in Pt-based alloys. The HR-TEM images of individual nanoparticles show that the particles are highly crystalline. The mean diameters of Pt-Ni, Pt-Co and Pt-Fe nanoparticles was measured and determined to be 6.62 nm ± 0.80 nm (standard deviation), 6.52 nm ± 0.81 nm and 6.38 nm ± 0.89 nm, respectively. The observed nanoparticles are concluded to be bimetallic Pt alloys; exhibiting uniform size and morphology, a narrow size distribution and good crystallinity.

**Figure 2.**
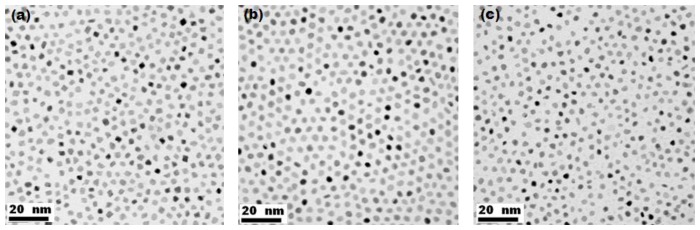
Bright-field TEM images of (**a**) PtNi (**b**) PtCo and (**c**) PtFe nanoparticles as above, but using OA as the surfactant in place of OAm in BE.

Bright-field TEM images of nanoparticles synthesized by replacing OAm with OA, also exhibiting uniform size, narrow size distribution, good dispersion and uniform morphology, are displayed in Figure 2. The reduction conditions were similar to those used for nanoparticle synthesis using OAm. As revealed by TEM images shown in Figure 1 and Figure 2, the particles did not require any size-selection processing to ensure uniformity. The reaction parameters and morphologies deduced from TEM observations of the as-prepared alloy nanoparticles under these reduction conditions are summarized in Table 1.

**Figure 3.**
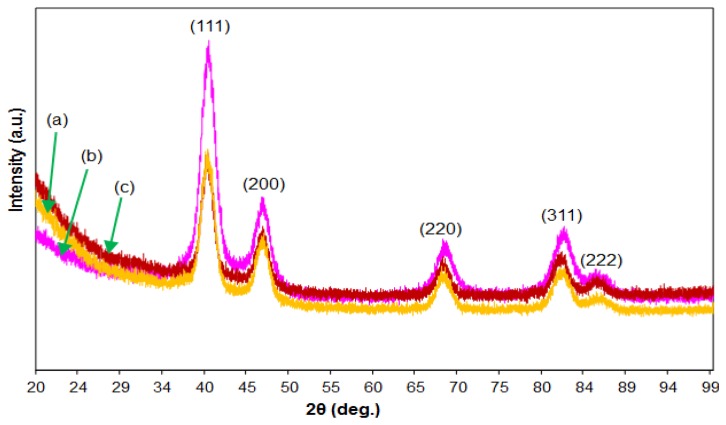
XRD patterns of PtM nanoparticles: (**a**) PtNi, (**b**) PtCo and (**c**) PtFe. Samples were deposited on an XRF microfine mylar polyester film on a flat sample holder.

Figure 3 shows XRD spectra from the as-prepared nanoparticle products. The positions and relative intensities of all the diffraction peaks of individual alloys can be indexed to an FCC phase. For all the patterns, the peaks in the range 38°–41°, 45°–48°, 66°–68° and 81°–83° correspond to the (111), (200), (220), (311) and (222) reflections of PtM binary alloys, confirming the successful synthesis of Pt-based solid solution as shown in electron diffraction patterns. No other diffraction peaks were identified, further demonstrating that only a characteristic single phase of FCC PtM exists in all the individual products.

The characteristics of the bimetallic nanoparticles shown in Figure 1, Figure 2 and 3, namely the size, crystallography, shape and chemical composition, are the result of manipulation of the synthetic process. Each characteristic is considered individually.

- The *size* of the nanoparticles is uniform, with an average diameter between 6 nm and 7 nm for all nanoalloys (Pt-Co, Pt-Ni and Pt-Fe). This indicates that nucleation was rapidly followed by a preference for growth on existing seeds, rather than continued nucleation. The elevated reduction temperature resulted in rapid nucleation followed by growth, moderated by organic surfactants, leading to a uniform size.
- The individual nanoparticles are shown to be fully crystalline with the FCC crystal structure. The phase structure of the nanoalloys is thus likely to be platinum-based solid solution, considered further with regard to compositional analysis (below).
- The final shape of the nanoparticles is spherical or near-spherical. Shape is determined by nucleation (rapid in this case) and the thermodynamic and kinetic characteristics of growth. The FCC nanoparticles show isotropic growth with no preferential growth direction. The morphology which develops is thus mainly determined by the overall surface energy which is at a minimum for the spherical shape, which has the lowest surface area [35]. After initial nucleation, the growth kinetics is sluggish (at least one hour for completion of the reduction reaction and addition of metal atoms to the nucleated seeds) in spite of the elevated temperatures used.
- The chemical compositions establish the coexistence of Pt and M in all nanoparticles. The anticipated final composition ratio of Pt:M was in the range (1.5–2):1. The molar ratios are however as follows: Pt:Ni = 65.3:34.7, Pt:Co = 88.7:11.3 and Pt:Fe = 79.4:20.6. The results thus show that the concentration of Co and Fe are below the expected solute content of the final products, but the Pt:Ni ratio is consistent with the initial molar feed ratio. This suggests that, in spite of the elevated reduction temperature, strong reductant, high boiling-point solvent, and extended time of reduction, the Co and Fe precursor salts had not fully reduced at the end of one hour (a further increase in the reduction temperature, or prolonging the reaction time, or increasing the molar ratio of M above what is finally desired, is expected to improve this). At these relatively low solute concentrations, the phase structure is expected to be the FCC platinum-based solid solution, consistent with the crystal structure consideration above.

The rate of reduction of Pt and M precursors thus appears to have been similar only for Pt-Ni nanoalloys. According to recent work by Zhang and Fang, Ni has an extremely strong affinity for inter-diffusion with Pt and thereby for formation of a binary nanoalloy [36]. In the absence of any evidence of ordering in EDPs, the Pt-Ni nanoparticles, like the Pt-Co and Pt-Fe nanoparticles, are concluded to form random FCC solid solutions.

The difference in the rate of reduction between different precursor chemistries is a strong argument for the dominance of the role of chemistry in the reaction. The role of the surfactants in growth kinetics is however also important: although the surfactants were employed primarily to stabilize the growth of nanoparticles, they may play a vital role in directing the crystal growth process. The use of a mixture of more than one or two surfactants may provide selective adsorption on distinct crystallographic facets of the growing crystals, leading to distinct crystallographic growth directions. For this reason, we investigated the effect of different surfactants on the final morphology. In Figure 1 it can be seen that selective binding of the employed surfactants resulted in the creation of mostly near spherical nanoparticles, since there is no obvious development of well-defined nanoparticle shapes. When OAm was replaced with OA, Pt-Ni nanoparticles were observed to adopt nearly cubic morphologies whereas Pt-Fe and Pt-Co nanoparticles did not show any significant change in shape. The development of crystal facets in Pt-Ni nanoparticles is thus sensitive to the type of surfactant employed, although all three nanoalloys appear to favour isotropic growth.

## 4. Conclusions

The use of multimetallic nanoparticles in biosensing has enormous potential provided that the nanoparticles can be synthesized reproducibly. The synthetic protocol is the sensitive link in the synthesis-structure-property chain, as it sensitively determines the ultimate nanoparticle size, shape and composition; and hence the nanoparticle properties. The importance of biosensing adds weight to the necessity for a robust method of production for nanoparticles. We present such a production protocol, which we have established by a systematic investigation of the synthesis of Pt-M nanoparticles, opening the way to development of highly sensitive bimetallic nanoalloy bioassay sensors.

## Figures and Tables

**Table 1.** Reaction parameters for the synthesis of highly monodispersed PtM alloy nanoparticles in the in the solvent BE.

**PtM**	**Surfactants**	**Solvent**	**Reducing Agent**	**Temperature (°C)**	**Time (min)**	**Shape**
PtNi	OAm, TOA, ODA	BE	TBAB	220	60	Spherical
PtCo	OAm, TOA, ODA	BE	TBAB	220	60	Near spherical
PtFe	OAm, TOA, ODA	BE	TBAB	260	60	Nearly spherical, lozenge
PtNi	OA, TOA, ODA	BE	TBAB	220	60	Nearly cubic
PtCo	OA, TOA, ODA	BE	TBAB	220	60	Nearly spherical, lozenge
PtFe	OA, TOA, ODA	BE	TBAB	260	60	Nearly spherical, less regular
